# Supplementary material for: In Vitro Microbiological and Drug Release of Silver/Ibuprofen Loaded Wound Dressing Designed for the Treatment of Chronically Infected Painful Wounds
Source: Antibiotics (Basel). 2021 Jul 2;10(7):805. doi: 10.3390/antibiotics10070805 (PMC8300664; doi:10.3390/antibiotics10070805)
Supplement: Supplementary file 1 [file antibiotics-10-00805-s001.zip › antibiotics-1267022-supplementary.pdf]

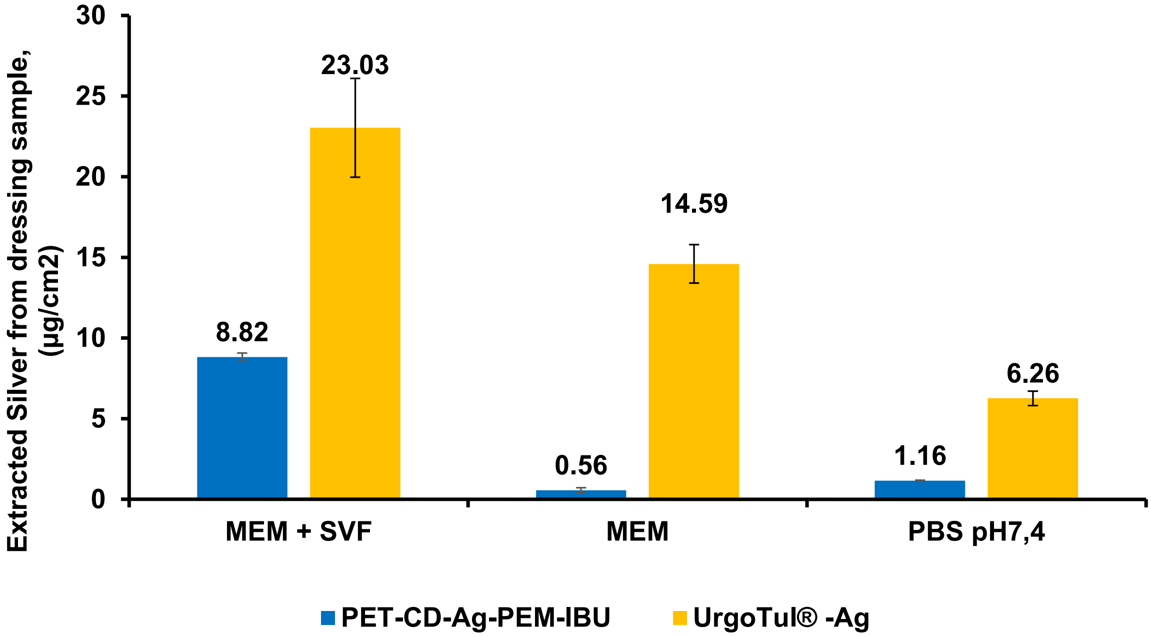

Figure S1. Comparison of the amount of silver extracted on the PET-CD-Ag-PEM-IBU (initial silver loading – 210 µg/cm²) and UrgoTul®-Ag (initial silver loading 350 µg/cm²) dressings after 24 hours stirring (80rpm) at 37 °C, in different extraction media.
